# Effects of Time Point Measurement on the Reconstruction of Gene Regulatory Networks

**DOI:** 10.3390/molecules15085354

**Published:** 2010-08-04

**Authors:** Wenying Yan, Huangqiong Zhu, Yang Yang, Jiajia Chen, Yuanyuan Zhang, Bairong Shen

**Affiliations:** Center for Systems Biology, Soochow University, 1st Shizi Street, Suzhou, Jiangsu 215006, China

**Keywords:** dynamic Bayesian networks, time points, gene regulatory network, network statistics, network reconstruction

## Abstract

With the availability of high-throughput gene expression data in the post-genomic era, reconstruction of gene regulatory networks has become a hot topic. Regulatory networks have been intensively studied over the last decade and many software tools are currently available. However, the impact of time point selection on network reconstruction is often underestimated. In this paper we apply the Dynamic Bayesian network (DBN) to construct the *Arabidopsis* gene regulatory networks by analyzing the time-series gene microarray data. In order to evaluate the impact of time point measurement on network reconstruction, we deleted time points one by one to yield 11 distinct groups of incomplete time series. Then the gene regulatory networks constructed based on complete and incomplete data series are compared in terms of statistics at different levels. Two time points are found to play a significant role in the *Arabidopsis *gene regulatory networks. Pathway analysis of significant nodes revealed three key regulatory genes. In addition, important regulations between genes, which were insensitive to the time point measurement, were also identified.

## 1. Introduction

Most biological networks, such as gene regulatory networks, protein-protein interaction networks and metabolic networks, are known to be complex and dynamic systems. However, many gene expression data in current microarray databases are static, which can hardly describe the life phenomenon well. Fortunately, time series gene microarray data, which contains the temporal information, could help with the dynamic network reconstruction, as is indicated in the gene knock-out experiments by Geier *et al.* [1]. In those experiments, the smaller the time interval is, the more accurate the result becomes. Accordingly, more data and costs are required. However, it is not desirable to make the interval too small, since the experiment data would be far more than enough when it comes to numerous gene observations.

Recently, many popular methods of gene regulatory network reconstruction were developed, including Boolean networks, multiple regression analyses [1], differential equations [2,3], mutual information [4,5], Bayesian networks (BNs) [6], *etc*. A Boolean network is a simple model that is suitable for qualitative research. The differential equations method, which models the gene network from an accurate mathematical point of view, lacks anti-noise ability and robustness. Researchers now pay more attention to Bayesian networks, including the static Bayesian network and the dynamic Bayesian network (DBN). The static Bayesian network, in which nodes represent random variables, models static probabilistic dependency relations among genes from its expression data with noise [6]. Although the method is considered to be effective, it constrains the network to be acyclic, which is contrary to the situation of real gene networks that have cyclic regulatory pathways such as feedback loops. Hence, DBN is a more promising choice for handling time series microarray data since it can construct cyclic pathways and describe feedback information of a system. The method represents a directed graphical model of a stochastic process. So far, some models are proposed based on the probability models of the dynamic Bayesian network model, such as the discrete model [7,8], vector autoregressive regression [9], the hidden Markov model [10,11,12,13], and so forth. The gene regulatory networks in this paper were constructed using a DBN approach with *Arabidopsis* time series gene microarray data. However, for the reconstruction of a gene regulatory network, two related issues are still unresolved. Firstly, the effect of time point measurements on the reconstruction of gene regulatory networks, such as the number of time points, and the measurement intervals, remain to be explored. Secondly, what kind of properties of the constructed network are robust and less sensitive to the time point measurements, i.e. what kind of properties obtained from the constructed networks are more credible, even when the time point measurement is not enough. To answer these two questions would be very helpful for the design of time course data measurements and the application of gene regulatory networks constructed with time series data. In this work, the reconstruction of the Arabidopsis gene regulatory network was taken as a case study to answer the above questions.

## 2. Experimental

### 2.1. Data and software

The data were derived from the microarray experiments performed in the laboratory of Smith (Edinburgh, UK) [14]. *Arabidopsis* were cultivated to growth stage 3.90 (Rosette growth complete) [15] and labeled for leaf harvesting. It involved sampling leaves at 11 different time points: 0, 1, 2, 4, 8, 12, 13, 14, 16, 20, and 24 h (the 24 h time point is a repeat of the 0 h one), where 0 h is the onset of dark and 12 h is the onset of light. The data are available in the NASCArrays database (http://affymetrix.arabidopsis.info/) [16] as experiment reference NASCARRAYS-60. We used R scripting to construct the *Arabidopsis* gene regulatory networks. The R package G1DBN was used to perform Dynamic Bayesian Network reconstruction [17,18]. Other R packages, such as sna, igraph, *etc *(http://mirrors.geoexpat.com/cran/) [17], were also used to analyze the network. All the related R scripts are listed and described in the online supplementary materials (http://www.sysbio.org.cn/Molecules2010_SupplementaryScripts.htm)

### 2.2. Method

#### 2.2.1. Dynamic Bayesian network method

DBN, in which a time factor is introduced, is an extension of the Bayesian network. More precisely, it uses time series data to construct causal connections among random variables and uses time lapse information to construct circular regulation [19]. The network structure can be denoted as *S* and *P*, where *S* is the structure of network and *P *is a set of conditional distribution on *S*. *S* represents a directed acyclic graph (DAG) and its nodes correspond to the time series dynamic variables. They can be defined as:

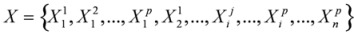
(1)
where: *X_i_^j ^*is the jth variable at time *i* and *x_i_^j^* is the value of jth variable at time *i,* 
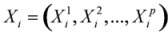
 is the vector composed by variables at time *i *and 
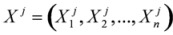
 is the vector composed by jth variable at all times.

The arc between two nodes of *S* represents the probabilistic relationship or causality between them. If there is an arc, the relationship of the two nodes will be conditional dependence. Then, the DBN model can be obtained:

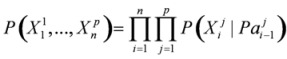
(2)
where *Pa^j^*_0_ = Ф,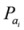 denotes the random variables that correspond to the parents of node *i*.

If the structure is unknown, the network will be constructed by some learning rules and relevant criterion, which can measure networks from observed data. Given an observed data set *D* of variables *X*, search for a network (*S*′,θ) such that it best matches the set *D*, where S′is the network structure and θ is parameters in network. Then, a score function can express how well it matched, that is, make the formula to be maximum:

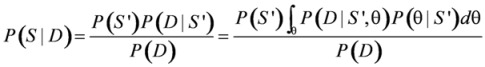
(3)


So far, compared to parameter learning, structure learning of DBN is much more difficult. In general, DBN structure learning approaches are transplanted and extended from the BN approaches and can be divided into two types. One is based on search and scoring method. At first, a primary network structure is given, and then edges are added or subtracted so that the model can be improved. Finally a network that best matches the dataset can be picked out. Another method is based on dependent relationships and uses statistical measurements to estimate the dependence among nodes and then construct a network based on the results. In the present work, we used the second method to construct gene networks from the *Arabidopsis* gene microarray data. 

#### 2.2.2. Network structure analysis

The network structure can be analyzed using different statistics based on the analysis of nodes, edges or the whole network. Various statistics could be analyzed for different goals. These statistics and other of the same type are commonly known as centrality measures, connectivity indices, and/or topological indices. The applications of these statistics cover drug molecular graphs [20,21], protein residue networks or protein interactions networks [22], host-parasite networks or cerebral cortex networks [23], social networks and internet [24] and other complex systems; including metabolic networks as one of the more interesting applications [25,26,27,28]. The basic statistics for nodes are degree, indegree and outdegree, which are defined as follows. Let *S = *(*V, E*’) be a directed network. *V* is the set of nodes and *E’* is the set of edges of network *S*, then the degree of a node *v *is the number of edges at node *v* [29], which belongs to *V*. The indegree or outdegree of a node *v * is the number of edges pointing to or out from node *v* in *S* [30]. Moreover, the diameter (*Dia*) is the longest shortest path of a network[30]. Here it is calculated by using a breadth-first search like method. Some of the other statistics adopted in this study are listed in Table 1. 

**Table 1:** Network statistics used in this paper.

Statistics	Definition	Descriptions
**Average degree *K *[29]**	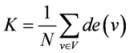	*de*(*v*): the degree of node *v*
		*N*: the number of nodes in network *S*
**Average path length *l*[30,32]**	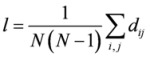	*d_ij_*: the shortest path between *v_i_* and *v_j_*
**Betweenness *B_v_*[33]**	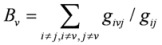	*g_ivj_*: the number of shortest paths from *i* to *j* that pass through a node *v*
		*g_ij_* : the number of shortest geodesic paths from *i* to *j*.
**Clustering coefficient CC [34]**	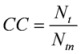	*_Nt_*_: number of closed triplets_
		*_Ntn_*_: number of connected triples of nodes_
**Centralization *Ce*( *S *) [35]**	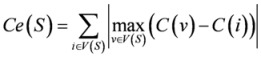	*C(v)*: the degree centrality for node *v *and 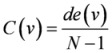
**Global efficiency of the network *E*[36]**	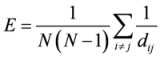	d_ij _: shortest path length
**Maximum vulnerability of the networks *Vu *[37]**	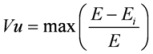	E: the efficiency of the network
		E_i _: the efficiency of the network without the node i and all edges connecting it with other vertices

#### 2.2.3. *Arabidopsis* gene regulatory networks reconstruction based on different time point deletion

To compare the effect of different time points on the reconstruction of network, groups of time series data should be used. However it is difficult to obtain abundant time point data in an experiment. Hence, we deleted the time points one by one to simulate the distinct groups of time series microarray data, which included 800 genes expression level at 11 time points, recording time 0, 1, 2, 4, 8, 12, 13, 14, 16, 20 and 24 h. Each time, we deleted one time point and constructed the gene regulatory network using the remaining time points. The networks were designated as G1, G2, G3 and G11. For instance, the network G1 was made up by time points 1, 2, 4, 8, 12, 13, 14, 16, 20 and 24. Additionally, the network with all the time points is denoted as G0. In order to express undulatory property of those statistics, for example, to find which statistics are more insensitive to the time points, we defined the relative diversity score of one statistics as follows:

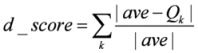
(4)
where *k*∈{0,1, 2,3,...,11}, *Q_k_* is a statistics of network *G_k_* and ave is the average of *Q_k_*. It is obvious that a low diversity score denotes a low undulatory property and here indicating the insensitivity to the time point measurement. 

## 3. Results and Discussion

### 3.1. The analysis of constructed Arabidopsis gene regulatory networks

**Figure 1:**
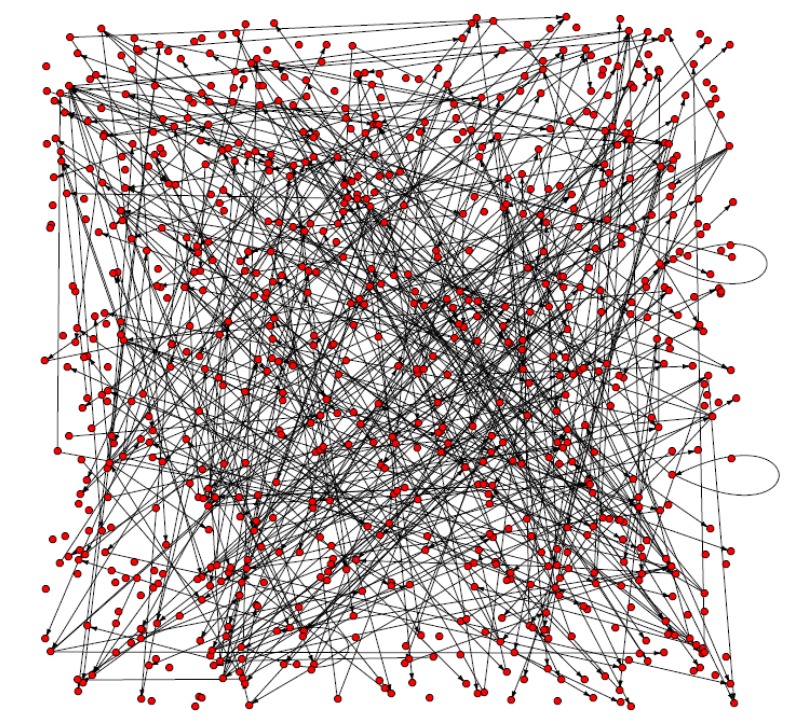
The directed network of *Arabidopsis* gene regulation. Red nodes represent genes and arcs represent the regulation between genes.

The *Arabidopsis* gene regulatory network built by the DBN method using the R software is shown in Figure 1. It consists of 800 genes and 447 gene regulations.

As shown in Table 2 the Clustering Coefficient is 0.0019 (<<1) and the centralization is 0.0093. These data indicate that the small community phenomenon was not obvious. The maximum vulnerability of the network is 0.0302. In Table 2 related statistics for *Arabidopsis* gene regulatory networks are listed. N0 is the node number whose degree is 0. Rn is the number of linear regulation between genes. The definition of other statistics can be found in Table 1.

**Table 2:** The statistics of Arabidopsis gene regulatory network.

*K*	*Dia*	*l*	*N0*	*Rn*	*E*	*Vu*	*CC*	*Ce*
**1.1175**	12	3.0467	306	447	0.0013	0.0302	0.0019	0.0093

**Figure 2:**
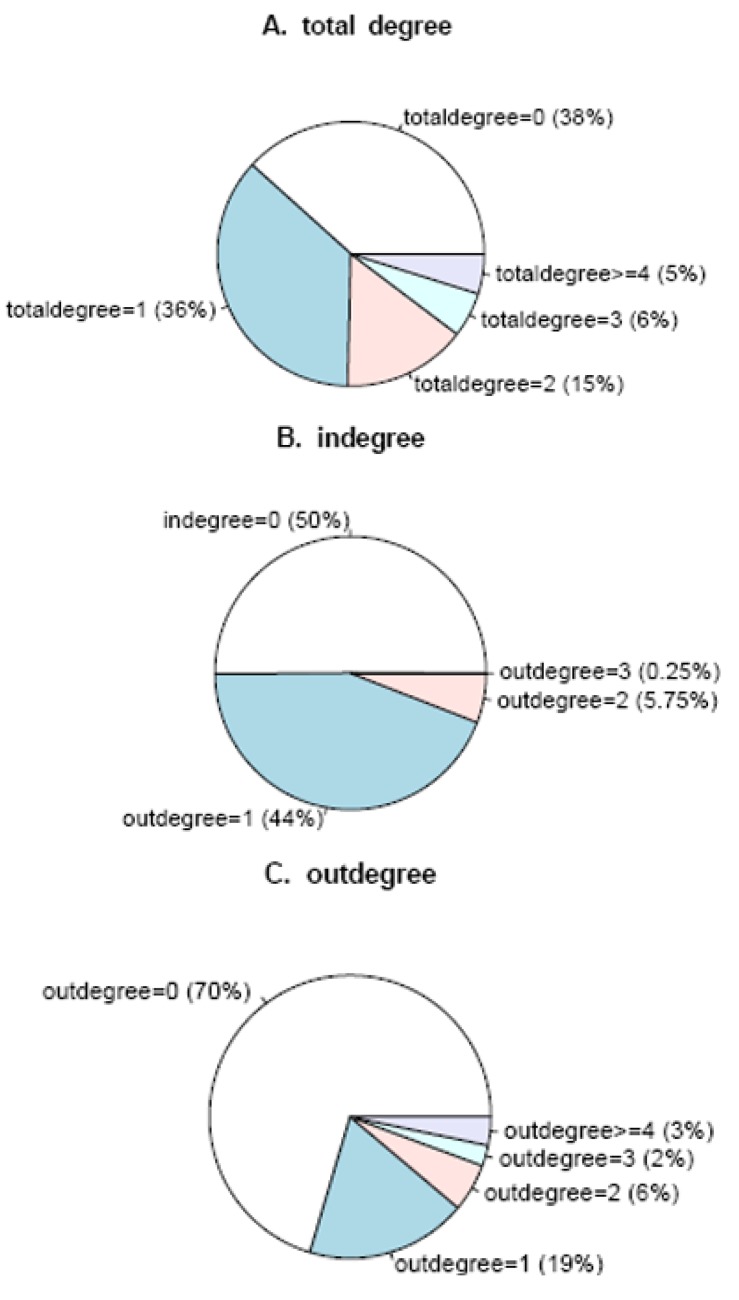
The degree of nodes in the *Arabidopsis* gene regulatory network. The three pie charts A, B and C denote outdegree, indegree, and total degree separately.

The distribution of degree of nodes in the *Arabidopsis* gene regulatory network is shown in Figure 2. 

It can be seen that over half the genes have regulatory relationships with others. About 36% of the nodes’ degree is 1, while about 5% of them are equal or greater than 4. That is to say 39 genes have regulation relationships with no less than four other genes. The gene with the maximum degree is disproportionating enzyme 2 (AT2G40840). It encodes a cytosolic protein during transglucosidase and amylomaltase activity, which suggests an essential role of the pathway carbohydrate metabolism in leaves at night [31]. Thus, most genes in the *Arabidopsis* gene regulatory network regulate or are regulated by other genes. The betweeness of gene nodes in the network was also calculated and the top-forty nodes were picked up. These genes were then mapped to KEGG database and 21 enriched pathways were identified and three key genes, At2g21330, At1g43670 and At2g29690, were observed to participate in most of these pathways.

The three genes are all significant in life progress of *Arabidopsis* and corresponding proteins in other species also have the similar important biological functions. They participate in fundamental metabolic pathways. Both At1g43670 and At2g21330 are involved in the carbohydrate metabolism: D-fructose-1,6-bisphosphate 1-phosphohydrolase (At1g43670) hydrolyzes the fructose-1,6-bisphosphate (F-1,6-P2) to fructose-6-phosphate (F-6-P) and inorganic phosphate; fructose-bisphosphate aldolase (At2g21330) catalyzes an aldol cleavage and its reversible aldol condensation of fructose-1,6-bisphosphate. Anthranilate synthase (At2g29690) takes part in the amino acid metabolism and is a key enzyme in the synthesis of tryptophan (Trp), indole-3-acetic acid, and indole alkaloids. O three genes, AT3g01920 (which encodes the yrdC family of proteins) AT3g57600 (encodes a member of the DREB subfamily A-2 in ERF/AP2 transcription factor family that responds to various types of biotic and environmental stress [38]) and AT1G51110 were found to have loops in the network. 

### 3.2. Identification of network statistics insensitive to time points measurement

Several network statistics of the 12 networks were calculated and are shown in Table 3. To find the network statistics that do not change much with different time points, we computed the diversity score of those statistics. From Table 3, it shows that diversity score of the average degree *K* (*p < 0.05*) and the number of regulations *Rn *(*p < 0.05*) are relatively small, while the diversity score of centralization *Ce* (*p < 0.05*) is larger. This indicated that average degree *K* and the number of linear regulation between genes *Rn* are less sensitive to time points and the centralization are sensitive to time points.. Therefore, gene regulatory networks based on these properties are more robust since they will not vary with time point measurements.

**Table 3:** Statistics values in 12 networks.

*Network*	*K*	*Dia*	*l*	*Ce*	*Rn*	*E*	*Vu*
**G0**	1.1175	12	3.0467	0.0093	447	0.001258	0.0302
**G1**	0.9750	10	2.4462	0.0101	390	0.000944	0.0397
**G2**	0.8725	6	1.6998	0.0095	349	0.000726	0.0499
**G3**	0.9175	6	1.9530	0.0076	367	0.000849	0.0366
**G4**	0.9525	11	2.3965	0.0101	381	0.000859	0.0602
**G5**	0.9625	5	1.8720	0.0289	385	0.000919	0.0809
**fG6**	0.9425	7	2.0107	0.0076	377	0.000811	0.0344
**G7**	0.8475	10	2.5515	0.0083	339	0.000804	0.0396
**G8**	0.9250	7	2.4457	0.0082	370	0.000892	0.0472
**G9**	0.8625	7	2.2134	0.0139	345	0.000784	0.0590
**G10**	0.9200	7	2.0985	0.0239	368	0.000863	0.0728
**G11**	0.9500	5	1.7365	0.0126	380	0.000806	0.0466
**ave**	0.9371	7.7500	2.2059	0.0125	374.8300	0.000876	0.0497
**d_score**	0.5785	2.7400	1.4780	4.6882	0.5800	1.07808	2.5885

In Table 3, each column is one set of statistics of the networks and each row represents all the statistics of one network. The two bottom rows illustrate the average and the relative diversity score of statistics in all 12 networks.

### 3.3. Comparison of the influence of different time points on the networks reconstruction

The maximum vulnerability is a valid statistic based on the whole network. It quantifies the maximum loss if one node is deleted from the network. The larger the value of the maximum vulnerability is, the less stable the network becomes. The maximum vulnerabilities of G5, G10, G4 and G9, rank among the top-four maximum vulnerabilities (Table 3). These networks get more loss than the left networks in performance because of the deletion of time point 8, 20, 4 and 16.

**Figure 3:**
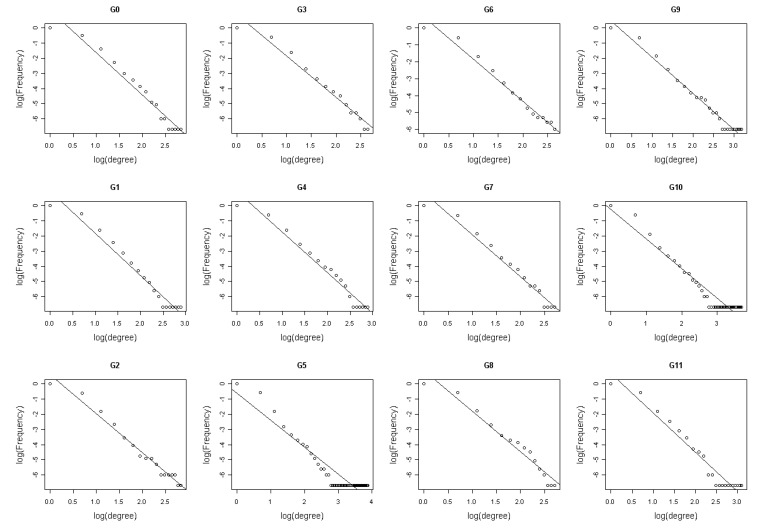
The degree logarithmic distribution for 12 networks (G0-G11). Most of them fit power-law distribution well.

**Figure 4:**
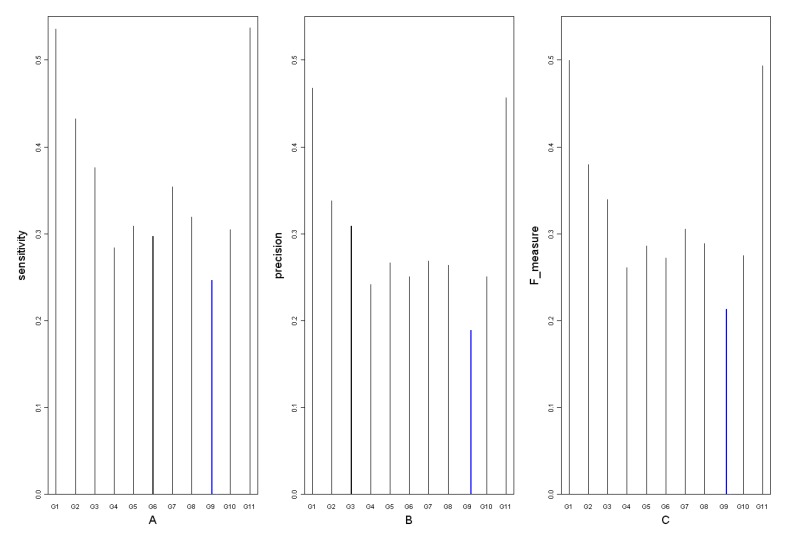
A is sensitivity of 11 time point removing networks with G0 as the standard network. B is precision of 11 networks and C shows F-measure.

In order to get the degree circumstances of the 12 networks, degree logarithmic distributions are considered (Figure 3). It is noteworthy that all of them fit the power-law distribution, which is a characteristic of scale-free networks [39]. Structures of the 12 networks are coherent on the whole. This may suggest that *Arabidopsis* gene regulatory networks deduced from time series microarray data are robust. Goodness of fit can be a crucial criterion to judge the robustness and stability of a network. From Figure 3 and Figure 4, the degree distribution of G0, G3, G6 and G8 fit better than other networks. It indicates that the deletion of the time points 2, 12 and 14 may not influence the networks’ degree distribution property significantly, or the networks are robust for these time points. Hence, the time points 2, 12 and 14 could be less important for the network reconstruction. On the other hand, distributions of G5, G9, G10 and G11 do not fit the power-law as well as others and their corresponding time points 8, 16, 20 and 24 are significant in the network degree properties. Network G0 is regarded as the standard network and the remaining 11 networks’ sensitivity and precision can then be obtained. The definitions of sensitivity and precision [40] are listed in Table 4.

**Table 4:** The definition of sensitivity, precision and F-measure.

Measurement	Definition		Descriptions
sensitivity	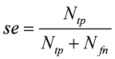		*N_tp_*: number of true positives *N_fn_*: number of false negatives *N_fp_*: number of false positives
precision	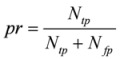		
F-measure	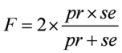		

True positive: existent regulation correctly diagnosed as existent; False positive: nonexistent regulation wrongly identified as existent; True negative: nonexistent regulation correctly diagnosed as nonexistent; False negative: existent regulation wrongly identified as nonexistent.

The values of sensitivity, precision and F-measure are all calculated for the eleven networks, and shown in Figure 4. The fact that all the values are not too large, suggests that almost every time point may have a considerable contribution to the network structure. By comparative analysis, we found that the sensitivity, precision and F-measure of G9, G4, G6 and G10 are smaller than those of the others. This shows that many regulations in those four networks are not recognized correctly just because of the deletion of these time points. By contrast, the three values of G1, G11, G2 and G3 are much larger, which means that regulations in these networks didn’t change much though they lack a time point. 

Combining the maximum vulnerability, degree distribution, sensitivity, precision and F-measure data of these eleven networks, the time points 16 (G9) and 20 (G10) are found to play a significant role in the *Arabidopsis* gene regulatory networks. Hence these time points should not be neglected for both the network reconstruction and biological experiments. In the same way, the time point 2 (G3) is found to be not as important as others.

Moreover, we evaluated the impact of time period on network construction by deleting two adjacent time points. The networks G2_3 and G9_10 were reconstructed by the data without two points, that is, 1h and 2h and 16h and 20h.

**Figure 5:**
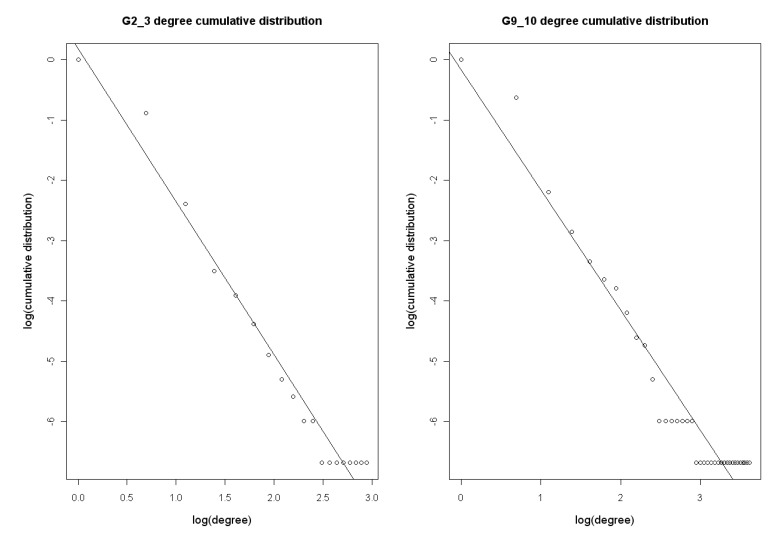
The degree logarithmic distribution for G2_3 and G9_10.

**Figure 6:**
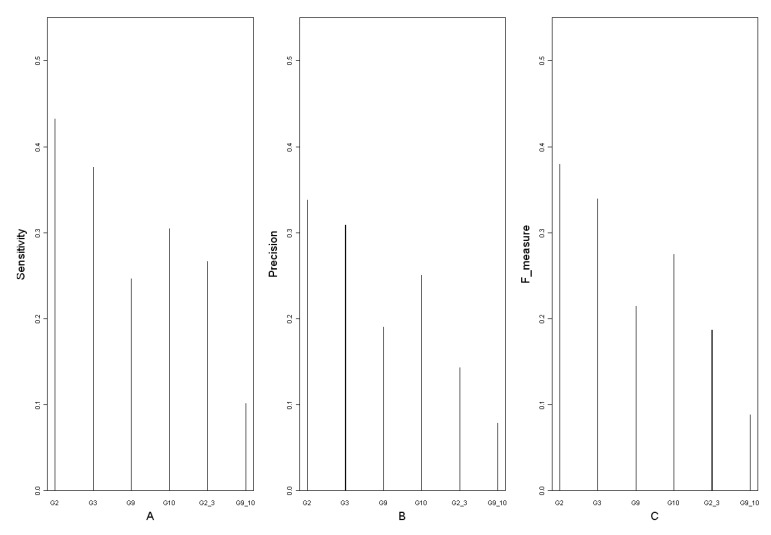
A is sensitivity of G2, G3, G9, G10, G2_3 and G9_10 with G0 as the standard network. B is precision of these 6 networks and C shows F-measure.

As is shown in Figure 5 and Figure 6, the deletion of two adjacent time points damages the constructed network, especially in the case of G9_10. 

Its maximum vulnerability is 0.0881, larger than the other networks (G0, G1, G2, G3, G4, G5, G6, G7, G8, G9, G10, G11, and G2_3). However the statistics such as maximum vulnerability (0.0769) and sensitivity of network G2_3 are not as significant as G9_10’s. This indicates that the period between 1h and 2h maybe not so important as 16h and 20h to the reconstruction of network.

**Figure 7:**
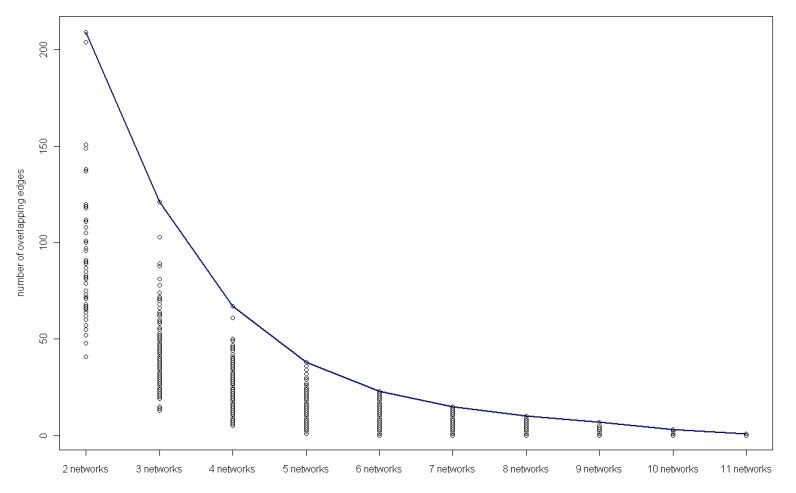
The number of overlapping edges in different networks.

### 3.4. Detection of key regulatory modules

On further analysis, occurrence of gene regulations in 12 networks should be considered. From Figure 7, the overlapping edges in the networks become less with the number of networks., and it is observed that four key gene regulations appear in 11 networks and ten in 10 networks (Table 5 and Table 6), showing that these regulations are insensitive with time, *i.e.* they happen during the most time in the experiment and they should be significant regulations in a sense. On the other hand, from these two tables, we can find that gene regulations were absent frequently in network G9 than in other networks, which is in agreement with the previous sensitivity and precision analysis. Gene regulations and signals in this time period are much more important and should be sampled more densely. 

**Table 5:** Four common gene regulations among 11 different networks.

Predictor	Target	Networks with the regulation	Network without the regulation
At1g77510	At1g17430	G0, G1, G2, G3, G5, G6, G7, G8, G9, G10, G11	G4
At3g02720	At2g30010	G0, G1, G2, G3, G4, G5, G6, G7, G8, G9, G10	G11
At5g06280	At1g77510	G0, G1, G2, G3, G4, G5, G6, G7, G9, G10, G11	G8
At5g58870	At5g38510	G0, G1, G2, G3, G4, G5, G6, G7, G8, G10, G11	G9

**Table 6:** Ten common gene regulations among 10 different networks.

Predictor	Target	Networks with the regulation	Network without the regulation
At1g01250	At4g16780	G0, G2, G3, G4, G5, G7, G8, G9, G10, G11	G1, G6
At1g36390	At4g09570	G0, G1, G4, G5, G6, G7, G8, G9, G10, G11	G2, G3
At1g07180	At3g01060	G0, G1, G2, G3, G5, G6, G7, G8, G9, G11	G4, G10
At1g07180	At5g35970	G0, G1, G2, G3, G5, G6, G7, G8, G9, G11	G4, G10
At3g5490	At3g10720	G0, G1, G2, G4, G5, G7, G8, G9, G10, G11	G3, G6
At5g40890"	At3g11710	G0, G1, G2, G3, G4, G6, G7, G8, G10, G11	G5, G9
At5g56900	At4g02380	G0, G2, G3, G4, G5, G6, G7, G8, G9, G10	G1, G11
At5g56900	At5g66920	G0, G1, G2, G3, G4, G6, G7, G8, G10, G11	G5, G9
At1g51110	At3g12760	G0, G1, G2, G3, G4, G5, G6, G7, G8, G10	G9, G11
At2g40890	At4g35090	G0, G1, G2, G3, G5, G6, G7, G8, G10, G11	G4, G9

## 4. Discussion and Conclusions

In the systems biology era, it has become necessary to study the dynamic behavior of a biological network with the time course data for a correct understanding of biological systems [41]. The measurement of time course data will become more and more popular, especially with the development of next generation sequencing technologies, which make the measurement of time course data cheaper and easier than ever before. However, to the best of our knowledge, until now, few works focused on the effect of time point measurements on the reconstruction of biological networks were reported. In this paper, the gene regulatory networks based on *Arabidopsis* time series data were constructed, and then the effect of the time point measurements on the network reconstruction was investigated. 

We have proposed a novel method to detect the effects of time point measurements, *i.e.* reconstruction of networks based on the deletion of different time points and then comparison of networks statistics at three different levels: degree, edges and networks. The time point deletion method can help us to detect the importance of different time points, to find the robust network properties and to identify key biological modules which are insensitive to time point measurement. According to our analysis, the network statistics such as the average degree (*K*) and the number of linear regulation between genes (*Rn*), are less sensitive to time point measurement, indicating that these statistics are more meaningful than others when even the time point measurement may not be enough. With our time point deletion method, we found that the time points 16 (G9), 20 (G10) in the *Arabidopsis* time course data are more important for the correct reconstruction of the *Arabidopsis *biological network, while the time point 2 (G3) is less important. In addition we also identified key biological regulations by the comparison of different time point deletion data sets.

The method proposed in this paper is based on the assumption that the networks statistics are more comparable if they were generated by the same network reconstruction method. We take the network G0 based on all time points as the standard network. Moreover, there are no perfectly correct networks that can be considered as the golden-standard reference. Of course, there are some other choices, such as take the independent network as the golden-standard network to validate. Further research should be done for this purpose. We could consider other network construction methods based on the time-series gene microarray data to validate the result, such as the reconstruction method by integrating several time course datasets [42]. 

## References

[1] Kato M., Tsunoda T., Takagi T. (2000). Inferring genetic networks from DNA microarray data by multiple regression analysis. Genome Inform. Ser. Workshop Genome Inform..

[2] Chen T., He H.L., Church G.M. (1999). Modeling gene expression with differential equations. Pac. Symp. Biocomput..

[3] de Hoon M.J., Imoto S., Kobayashi K., Ogasawara N., Miyano S. (2003). Inferring gene regulatory networks from time-ordered gene expression data of Bacillus subtilis using differential equations. Pac. Symp. Biocomput..

[4] Basso K., Margolin A.A., Stolovitzky G., Klein U., Dalla-Favera R., Califano A. (2005). Reverse engineering of regulatory networks in human B cells. Nat. Genet..

[5] Liang S., Fuhrman S., Somogyi R. (1998). Reveal, a general reverse engineering algorithm for inference of genetic network architectures. Pac. Symp. Biocomput..

[6] Friedman N., Linial M., Nachman I., Pe'er D. (2000). Using Bayesian networks to analyze expression data. J. Comput. Biol..

[7] Ong I.M., Glasner J.D., Page D. (2002). Modelling regulatory pathways in E. coli from time series expression profiles. Bioinformatics.

[8] Zou M., Conzen S.D. (2005). A new dynamic Bayesian network (DBN) approach for identifying gene regulatory networks from time course microarray data. Bioinformatics.

[9] Opgen-Rhein R., Strimmer K. (2007). Learning causal networks from systems biology time course data: an effective model selection procedure for the vector autoregressive process. BMC Bioinformatics.

[10] Beal M.J., Falciani F., Ghahramani Z., Rangel C., Wild D.L. (2005). A Bayesian approach to reconstructing genetic regulatory networks with hidden factors. Bioinformatics.

[11] Perrin B.E., Ralaivola L., Mazurie A., Bottani S., Mallet J., d'Alche-Buc F. (2003). Gene networks inference using dynamic Bayesian networks. Bioinformatics.

[12] Rangel C., Angus J., Ghahramani Z., Lioumi M., Sotheran E., Gaiba A., Wild D.L., Falciani F. (2004). Modeling T-cell activation using gene expression profiling and state-space models. Bioinformatics.

[13] Wu F.X., Zhang W.J., Kusalik A.J. (2004). Modeling gene expression from microarray expression data with state-space equations. Pac. Symp. Biocomput..

[14] Smith S.M., Fulton D.C., Chia T., Thorneycroft D., Chapple A., Dunstan H., Hylton C., Zeeman S.C., Smith A.M. (2004). Diurnal changes in the transcriptome encoding enzymes of starch metabolism provide evidence for both transcriptional and posttranscriptional regulation of starch metabolism in Arabidopsis leaves. Plant Physiol..

[15] Boyes D.C., Zayed A.M., Ascenzi R., McCaskill A.J., Hoffman N.E., Davis K.R., Gorlach J. (2001). Growth stage-based phenotypic analysis of Arabidopsis: A model for high throughput functional genomics in plants. Plant Cell.

[16] May S. NASC's International Affymetrix Service. http://affymetrix.arabidopsis.info/.

[17] The Comprehensive R Archive Network http://mirrors.geoexpat.com/cran/.

[18] LÃ¨bre S. (2009). Inferring Dynamic Genetic Networks with Low Order Independencies. Stat. Appl. Genet. Mol. Biol..

[19] Murphy K., Mian S. (1999). Modelling Gene Expression Data using Dynamic Bayesian Networks.

[20] Carlson J.M., Chakravarty A., Khetani R.S., Gross R.H. (2006). Bounded search for de novo identification of degenerate cis-regulatory elements. BMC Bioinformatics.

[21] Todeschini R., Consonni V., Mannhold R., Kubinyi H., Timmerman H. (2000). Handbook of Molecular Descriptors.

[22] Gonzalez-Diaz H., Gonzalez-Diaz Y., Santana L., Ubeira F.M., Uriarte E. (2008). Proteomics, networks and connectivity indices. Proteomics.

[23] González-Díaz H., Munteanu C.R. (2010). Topological Indices for Medicinal Chemistry, Biology, Parasitology, Neurological and Social Networks.

[24] Stefan B., Heinz Georg S. (2003). Handbook of Graphs and Networks: From the Genome to the Internet.

[25] Mrabet Y., Semmar N. (2010). Mathematical methods to analysis of topology, functional variability and evolution of metabolic systems based on different decomposition concepts. Curr. Drug Metab..

[26] Chou K.C. (2010). Graphic rule for drug metabolism systems. Curr. Drug Metab..

[27] Gonzalez-Diaz H. (2010). Network topological indices, drug metabolism, and distribution. Curr. Drug Metab..

[28] Gonzalez-Diaz H., Duardo-Sanchez A., Ubeira F.M., Prado-Prado F., Perez-Montoto L.G., Concu R., Podda G., Shen B. (2010). Review of MARCH-INSIDE & complex networks prediction of drugs: ADMET, anti-parasite activity, metabolizing enzymes and cardiotoxicity proteome biomarkers. Curr. Drug Metab..

[29] Diestel R. (1997). Graph theory. Graduate Texts in Mathematics.

[30] West D. (1996). Introduction to Graph Theory.

[31] Chia T., Thorneycroft D., Chapple A., Messerli G., Chen J., Zeeman S.C., Smith S.M., Smith A.M. (2004). A cytosolic glucosyltransferase is required for conversion of starch to sucrose in Arabidopsis leaves at night. Plant J..

[32] Albert R., Barabasi A.-L. (2002). Statistical mechanics of complex networks. Rev. Mod. Phys..

[33] Freeman L.C. (1977). A set of measures of centrality based on betweenness. Sociometry.

[34] Wasserman S., Faust K. (1994). Social Network Analysis: Methods and Applications (Structural Analysis in the Social Sciences).

[35] Freeman L. (1979). Centrality in social networks: Conceptual clarification. Soc. Networks.

[36] Latora V., Marchiori M. (2001). Efficient behavior of small-world networks. Phys. Rev. Lett..

[37] Gol'dshtein V., Koganov G.A., Surdutovich G.I. (2004). Vulnerability and Hierarchy of Complex Networks. arXiv: preprint cond-mat/0409298.

[38] Riechmann J.L., Meyerowitz E.M. (1998). The AP2/EREBP family of plant transcription factors. Biol. Chem..

[39] Barabasi A.L., Bonabeau E. (2003). Scale-free networks. Sci. Am..

[40] Altman D.G., Bland J.M. (1994). Diagnostic tests. 1: Sensitivity and specificity. BMJ.

[41] Dzeroski S., Todorovski L. (2008). Equation discovery for systems biology: finding the structure and dynamics of biological networks from time course data. Curr. Opin. Biotechnol..

[42] Wang Y., Joshi T., Zhang X.S., Xu D., Chen L. (2006). Inferring gene regulatory networks from multiple microarray datasets. Bioinformatics.

